# Optimizing antiphospholipid antibody testing: a real-world analysis of appropriateness and resource utilization

**DOI:** 10.1007/s12026-025-09682-x

**Published:** 2025-09-16

**Authors:** Silvia Grazietta Foddai, Maria Infantino, Mariangela Manfredi, Francesca Pavia, Maurizio Benucci, Francesca Li Gobbi, Massimo Radin, Irene Cecchi, Alice Barinotti, Savino Sciascia

**Affiliations:** 1 University Center of Excellence on Nephrologic, Rheumatologic and Rare Diseases (ERKNet, ERN-Reconnet and RITA-ERN Member) with Nephrology and Dialysis Unit and Center of Immuno-Rheumatology and Rare Diseases (CMID), Coordinating Center of the Interregional Network for Rare Diseases of Piedmont and Aosta Valley, San Giovanni Bosco Hub Hospital, Turin, 10154 Italy; 2 Department of Clinical and Biological Sciences, University of Turin, Turin, Italy; 3 Immunology and Allergology Laboratory, S. Giovanni di Dio Hospital, Florence, Italy; 4 Rheumatology Unit, S. Giovanni di Dio Hospital, Florence, Italy

**Keywords:** APL antibodies, Appropriateness, Clinical queries

## Abstract

Efficient utilization of healthcare resources, including laboratory testing, is crucial for environmental sustainability and cost-effectiveness. The diagnosis of APS requires the presence of at least one clinical event (either an objectively confirmed thrombotic event and/or pregnancy complication) and detection of one or more aPL (lupus anticoagulant [LA], IgG/IgM anticardiolipin [aCL], and/or IgG/IgM anti-β2 glycoprotein-1 [aβ2GPI]). However, inappropriate requests for aPL tests contribute to unnecessary healthcare expenses and environmental impact. This study evaluates the appropriateness of aPL testing in a clinical setting. A retrospective analysis was conducted on 642 patients attending the San Giovanni Di Dio Hospital, Florence (11/2023–02/2024). Diagnostic suspicion underlying aPL test requests were classified as appropriate, inappropriate, or unevaluable using a scoring system based on clinical recommendations. Appropriateness assessment was performed independently by two researchers and reconciled with a third expert. Patient demographics, test results, and the specialty of the physicians ordering aPL were recorded and analyzed. Of the 642 queries, 36% were deemed appropriate, 42% inappropriate, and 22% unevaluable. Family physicians accounted for 53% of all test requests but exhibited the highest rate of inappropriate requests (44%). Rheumatologists, internal medicine physicians, and gynecologists demonstrated better adherence to recommendations (with 34%, 30%, and 18% of inappropriate requests, respectively). Only 4.9% of patients underwent comprehensive aPL testing per international standards (Sidney criteria). Among the 115 aPL-positive cases, multiple antibody positivity was more common in appropriate test requests. Inappropriate requests often stemmed from conditions without established links to APS, such as alopecia, hypercholesterolemia, and dysmenorrhea. A considerable proportion of aPL testing in routine practice lacks clinical justification, reflecting variability in guideline adherence across specialties. Inappropriate testing increases healthcare costs, specialist referrals, and environmental burdens. Improved education, adherence to diagnostic recommendations, and sustainable practices are critical to optimizing APS testing and resource utilization.

**Supplementary Information:**

The online version contains supplementary material available at 10.1007/s12026-025-09682-x.

## Introduction

Revised and rational resources utilization represent one of the main goals for environment control and preservation [1]. In this context, correct requests for laboratory testing is mandatory to reduce unnecessary utilization of natural resources (water, power, electricity, etc.), specialists’ examinations, and, in a wider view, healthcare costs. In the last 20 years, the number of subjects affected by autoimmune disorders rose due to a combination of increased testing sensitivity and methodology of detection and knowledge. Recent evidence also showed an interesting role of the environmental boost sustained by pollution [2–4]. Autoimmune antibodies can be detected in the blood years before disease abruption; therefore, their identification helps physicians to pin down patients in an early phase of the disease. For example, tests for anti-DNA antibodies can be positive many years before the diagnosis of SLE and tend to follow a predictable course, with a progressive accumulation of specific autoantibodies before the onset of the disease, while patients are still asymptomatic [5, 6]. At the same time, even the test with the highest specificity bears intrinsically the chance of false positivity. In this context, clinical judgment is fundamental to reduce unnecessary consultations, patient anxiety, misdiagnoses, misguided therapies, and an unnecessary cascade of insights, demanding specialized testing only when necessary. Nowadays, a wider spectrum of clinicians order autoantibodies testing, e.g., antinuclear antibodies (ANA) and antiphospholipid antibodies (aPL), including internists, dermatologists, nephrologists, oncologists, cardiologists, neurologists, gastroenterologists, otolaryngologists, ophthalmologists, gynecologists, and even family physicians. Recent studies have highlighted how ANA, an entry level testing in the rheumatologic field, is overly prescribed leading to unnecessary resource consumption [7]. This can be related to the broadening spectrum of ANAs observed in various diseases, including antiphospholipid syndrome (APS), rheumatoid arthritis, autoimmune liver disease, vasculitis, inflammatory bowel disease, and cancer [8]. This reasoning has even more meaning in the field of rare disorders, such as APS, in which the current estimated prevalence in the general population is less than 1:2000 [9]. APS is sustained by concurrent participation of different actors, such as endothelial, platelets, white blood cells, and complement [10–12]. All the above compete in determining disease’s clinical features that range from the most paradigmatic events, thrombosis and pregnancy morbidity to rarer manifestations such as heart valve disease, aPL nephropathy, and thrombocytopenia [12]. Diagnosis is clinical considering that diagnostic criteria are lacking, however for cohort harmonization, classification criteria have been released and revised [13]. In this context, however, aPL testing represents a useful tool to orientate physicians in case of high suspicion for the disease. As for therapeutic approach, long-life anticoagulation represents its cornerstone; however, antiaggregating treatments and immunomodulatory drugs can be used in selective cases. Testing for aPL should be driven by high clinical suspicion considering that it is not a primary test [14]. In this paper, we explored the prevalence of aPL in a clinical setting of patients suspected for APS and the appropriateness of its requests considering current recommendations [14, 15].

## Methods

Inpatients and outpatients attending the San Giovanni Di Dio Hospital, Florence, Italy, were retrospectively studied between 08/2023 and 10/2023 for the following parameters: aPL testing results; data on sex, age; clinical query sustaining antibodies determination; prescriber doctor specialty. If the diagnostic suspicion was missing, the patient was excluded from the study. The clinical queries/diagnostic suspicion underlying testing for aPL was assessed and codified as inappropriate (group 0), appropriate (group 1), or unable to be evaluated (group 2) as in accordance to ACR/EULAR 2023 criteria. The appropriateness of aPL test requests was independently assessed by two investigators (SGF and MI), each with at least 6 years of experience in managing patients with APS. A standardized scoring system was applied, assigning up to 4 points based on two components: (1) the clinical indication for testing and (2) the completeness of the clinical query. Completeness was defined as the presence of sufficient clinical detail to support the diagnostic suspicion. For example, a query such as “Patient with a history of recurrent early pregnancy losses” was considered complete, while “miscarriages” alone was deemed insufficient. Queries scoring ≥ 3 were classified as appropriate, and those scoring 0 as inappropriate. Queries receiving 1 or 2 points were reviewed by the initial evaluators together with a third expert (SS) to reach consensus. If the clinical query solely reported the note “control,” the scoring system was not applicable and the aPL testing request was considered “unable to be evaluated.” Domains and related variables applied to quantify aPL tests appropriateness are reported in Table 1. The aPL testing [anticardiolipin antibodies (aCL) IgG and IgM, anti-B2 glycoprotein I (aB2GPI IgG and IgM)] and anti-B2 glycoprotein I-Domain 1 were performed using QUANTA Flash assay (INOVA Diagnostics, Inc., San Diego, CA) on the BIO-FLASH instrument (Biokit SA, Barcelona, Spain), while lupus anticoagulant assay (LA) test was determined using ISTH recommendations [16]. Note: In this paper, we refer to subjects who tested positive for aCL (regardless of isotype), aβ2GPI (regardless of isotype), anti-D1, and LA as “tetra-positive.” We acknowledge that in the research field, the term “tetra-positive” has also been used to describe criteria aPL positivity in conjunction with anti-phosphatidylserine/prothrombin (aPS/PT) positivity. Our usage deviates from that convention and is based solely on the four specific aPL markers listed above. The cutoff value recommended is 20 CU/mL for either IgG or IgM aCL or aB2GPI. Data were retrieved by electronic charts of the hospital and anonymized before analysis; descriptive statistics were used to process information. The study was conducted in accordance with the Declaration of Helsinki.

**Table 1:** Domains and related variables applied to quantify aPL tests appropriateness

	Scoring criteria	Scoring range (0–3)
Clinical indication	Strong alignment with recommendations	3
	Partial alignment (e.g., non-classical APS features)	2
	Performed probably for research purpose; clinical manifestation potentially linked to APS	1
	No clear clinical indication	0
Query completeness	Sufficient detail provided	1
	Insufficient detail provided	0

## Results

### General characteristics

Up to 642 patients were considered eligible for study. Of them, 75.2% (483) were women, 24.8% (159) men, with a mean age of 52 (± 19) years, median age 53 years. Interestingly, only 32 subjects were tested for all three aPL criteria, accounting for 4.9% of the cohort. In detail, all patients were tested for solid aPL, while only in 32 cases LA determination was performed. Out of the 642 patients, 115 were aPL positive (17.9% of the whole cohort), of which 80% were females (92/115). Mean age of the aPL-positive cohort was 50 ± 18 years; median 49 years. When considering aPL positivity, most patients resulted single aPL positive 70.4% (81/115), while 17.4% presented double (20/115), 7.8% triple (9/115), and 4.4% tetra positivity (5/115). When looking at the aPL specificity, the most represented was aCL (79 IgG, 33 IgM), followed by aB2GPI (IgG 30, IgM 15) and LA (10). The aPL negative cohort included 527 patients, mainly women (391, 74%), with a mean age of 53 (± 19) years and a median of 54 years.

### Assessing request appropriateness taking into account completeness and reported diagnostic suspicion

**Fig. 1:**
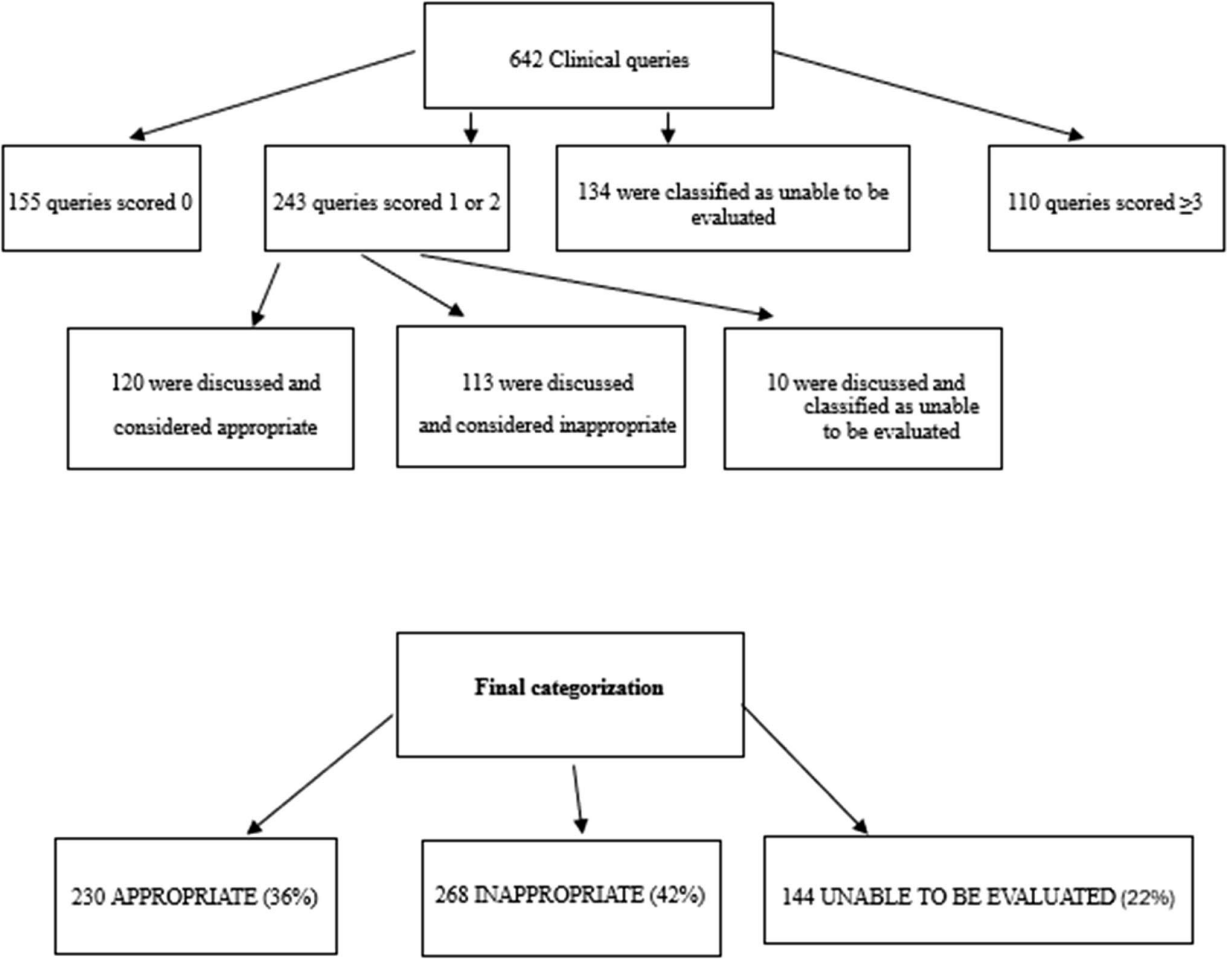
Flowchart of clinical queries evaluation

Considering the whole cohort (642), 110 queries scored ≥ 3, 155 scored 0, and 134 as unable to be evaluated. Two hundred forty-three clinical queries were scored 1 or 2 and were further evaluated. In detail, 120 were discussed and considered as appropriate, 113 were classified as inappropriate, and 10 as not able to be evaluated. In total, 230 queries were appropriate (36%), 268 inappropriate (42%), and 144 unable to be evaluated (22%). Complete flowchart is presented in Fig. 1. When considering prescription rates, family physicians resulted the most frequent prescribers (341/642, 53%) and scored for the highest rate of inappropriate requests (148/341, 44%) together with neurologists (11/24, 46%), hematologists (12/23, 52%), gastroenterologists (6/7, 86%), immunologists/allergologists (3/5, 60%), and pneumologists (2/3, 67%). Some specialists were present with a single query and most of them requested aPL testing in an inappropriate manner. As for appropriate testing, rheumatologists (65/130, 50%), internal medicine doctors (19/36, 53%), gynecologists (28/28, 71%), and nephrologists (5/8, 63%) scored the highest appropriate rate of aPL testing. Almost a quarter of all queries were not evaluable and among them (103/144, 71%) derived from family physicians’ requirements. Appropriate testing request according to doctor’s specialty is represented in Table 2.

**Table 2:** Appropriateness evaluation per prescription doctors

(Group type)	Requests evaluation		
	Inappropriate (0)	Appropriate (1)	Not evaluable (2)
aPL testing requests evaluation out of 642 total requests: *N* (%)	268 (42)	230 (36)	144 (22)
aPL testing requests evaluation for physician specialty: *N* (%)	Family physician: 148 (44)	Family physician: 90 (26)	Family physician: 103 (30)
	Rheumatologist: 44 (34)	Rheumatologist: 65 (50)	Rheumatologist: 21 (16)
	Internal medicine: 11 (30)	Internal medicine: 19 (53)	Internal medicine: 6 (17)
	Gynecologist: 5 (18)	Gynecologist: 20 (71)	Gynecologist: 3 (11)
	Neurologist: 11 (46)	Neurologist: 9 (37)	Neurologist: 4 (17)
	Hematologist: 12 (52)	Hematologist: 10 (43)	Hematologist: 1 (5)
	Nephrologist: 2	Nephrologist: 5	Nephrologist: 1
	Gastroenterologist: 6	Gastroenterologist: 1	Gastroenterologist: 0
	Endocrinologists: 3	Endocrinologists: 1	Endocrinologists: 2
	Immuno-allergologist: 3	Immuno-allergologist: 2	Immuno-allergologist: 0
	Pediatrician: 2	Pediatrician: 2	Pediatrician: 1
	Cardiologist: 2	Cardiologist: 2	Cardiologist: 1
	Pneumologist: 2	Pneumologist: 0	Pneumologist: 1
	Oncologist: 3	Oncologist: 0	Oncologist:
	Urologist: 2	Urologist: 0	Urologist: 0
	Transfusion medicine doctor: 1	Transfusion medicine doctor: 1	Transfusion medicine doctor: 0
	Emergency care doctor: 1	Emergency care doctor: 1	Emergency care doctor: 0
	Geriatrician: 2	Geriatrician: 0	Geriatrician: 0
	Radiotherapist: 2	Radiotherapist: 0	Radiotherapist: 0
	Anesthesiologist: 1	Anesthesiologist: 0	Anesthesiologist: 0
	General surgeon: 1	General surgeon: 0	General surgeon: 0
	Dermatologist: 1	Dermatologist: 0	Dermatologist: 0
	Physiatrist: 1	Physiatrist: 0	Physiatrist: 0
	Infectiologist: 1	Infectiologist: 0	Infectiologist: 0
	Sport medicine doctor: 1	Sport medicine doctor: 0	Sport medicine doctor: 0
	Clinical pathologist: 0	Clinical pathologist: 1	Clinical pathologist: 0
	Psychiatrist: 0	Psychiatrist: 1	Psychiatrist: 0

To note: Specialist requests are reported only as an absolute number and not in percentage if < 10  entries are present

#### Group 0: inappropriate requests

We grouped test requests by clinical category even when the prescriber’s specialty did not match the category indicated in the query field (e.g., a hematology-classified request submitted by a gynecologist). Across specialties, numerous aPL test requests were considered inappropriate. In oncology, 23 queries related to cancer, anticoagulation, and comorbidities were submitted by a range of prescribers. Although malignancy is a known prothrombotic condition, aPL positivity does not alter anticoagulation strategies, either pre- or post-cancer diagnosis, making testing generally unjustified in this context. In cardiology, two queries concerning aortic valve disease and tachycardia lacked any clinical connection to APS. Likewise, in dermatology, 12 queries related to alopecia, dermatitis, and cellulitis—submitted by multiple specialists—were deemed unrelated to APS and thus not appropriate indications for aPL testing.

Endocrinology-related requests (*n* = 36), primarily addressing diabetes, thyroiditis, and hypercholesterolemia, also lacked justification for aPL testing. Hypercholesterolemia and osteoporosis, though relevant in APS management, do not independently support testing in the absence of other criteria. Autoimmune thyroiditis may warrant research-based testing, but routine screening in thyroid disorders is not advised. In gastroenterology, 27 queries addressed hepatic abnormalities and gastrointestinal disorders. Although conditions like Budd-Chiari syndrome or HELLP may involve APS, elevated liver enzymes, bilirubin, or AMA positivity alone does not substantiate aPL testing. Similarly, testing in inflammatory bowel disease or celiac disease is not routinely indicated and may only be appropriate in research settings. In viral hepatitis, testing is discouraged due to potential transient aPL positivity secondary to infection.

In gynecology, 17 queries focused on various reproductive conditions, including amenorrhea, metrorrhagia, endometriosis, and menopause. These lacked evidence-based associations with APS, and aPL testing was not indicated. Likewise, conditions such as gestational diabetes, ovarian cysts, and fibroids do not align with APS pathophysiology. Sickle cell carriers, without other clinical features of APS, also do not require testing. Hematology-related queries (*n* = 26) included anemia, lymphadenopathy, and suspected hematologic malignancies. While APS can cause hemolytic anemia, anemia is a nonspecific finding, and testing without additional criteria is not supported. Testing was also inappropriate in cases of elevated D-dimer or fibrinogen, which are nonspecific inflammatory markers and may interfere with lupus anticoagulant detection. Coagulation factor mutations (e.g., factor V Leiden) may occasionally coexist with APS, but routine aPL screening is not warranted without suggestive clinical features.

Immunologic/allergic and infectious disease queries were rare (*n* = 3), involving anaphylaxis, nickel allergy, and HIV. None of these conditions supports routine aPL testing outside of a research context. Similarly, an internal medicine query regarding cardiac amyloidosis, while of academic interest, did not meet criteria for testing. In nephrology, two queries concerned proteinuria and congenital anomalies. Although APS nephropathy may present with proteinuria, isolated proteinuria does not justify screening. Neurology-related queries (*n* = 11) involved multiple sclerosis (MS) and myasthenia gravis. While aPL positivity has been noted in MS, especially under immunomodulatory therapy, testing has no diagnostic role once MS is established.

Other specialties showed similarly low appropriateness for aPL testing. In ophthalmology, otolaryngology, orthopedics, pulmonology, and urology (*n* = 1–5 queries per specialty), queries lacked association with APS. In rheumatology (*n* = 53), conditions such as ANA positivity, arthralgia, Raynaud’s phenomenon, and connective tissue diseases were commonly cited. Although these can co-occur with APS, none is specific. ANA is found in up to 13% of the general population, with increasing prevalence with age and overtime [17, 18]. Testing based solely on nonspecific rheumatologic symptoms or ANA positivity is not indicated. In rheumatologic conditions such as psoriatic arthritis, polymyositis, Raynaud syndrome, and suspected vasculitis, testing may be considered for research, but not for routine diagnosis. Anti-TNF therapy may induce aPL positivity, but this does not alter vascular risk and does not justify testing. Only one query cited treatment monitoring as the rationale; however, aPL levels rarely guide therapy after diagnosis.

Vascular medicine queries (*n* = 4) addressed atherosclerosis and bleeding symptoms. While APS can accelerate atherosclerosis, general screening is not recommended. Finally, in the “mixed” category (*n* = 39), broad, often unrelated symptom clusters (e.g., oral ulcers, shoulder periarthritis) further complicated assessment, and none clearly supported aPL testing. Overall, across disciplines, most test requests lacked a clinical rationale aligned with established APS criteria. All queries, reasons for testing, and assessment of appropriateness are detailed in Supplementary Table 1.

#### Group 1: appropriate requests

Several clinical queries were deemed appropriate for aPL testing. In oncology, one case involved prior aPL positivity - before cancer diagnosis - in a cancer patient, justifying retesting due to the increased prothrombotic state associated with malignancy. Dermatology queries addressed progressive ulcers and purpura—both recognized APS manifestations. In gynecology, 46 queries involved recurrent miscarriage, preeclampsia, IUGR, and postpartum hypertension, all linked to APS. Additionally, infertility and assisted reproduction in patients with thyroiditis or celiac disease supported testing for risk stratification or research.

In hematology, 49 queries involving thrombotic events, thrombocytopenia, hemolytic anemia, suspected coagulopathies, and monoclonal gammopathy were consistent with APS and justified testing. Neurological symptoms such as migraines with aura, optic neuritis, epilepsy, TIA, and stroke also supported testing, as did a case of Guillain-Barré syndrome.

Nephrology-related queries addressed chronic kidney disease and post-transplant evaluations, both appropriate due to potential APS nephropathy. A case of sudden sensorineural hearing loss, a rare but reported APS feature, also warranted testing. In rheumatology, 107 queries involving suspected or confirmed connective tissue diseases—including SLE, Sjögren’s syndrome, and systemic sclerosis—were aligned with current guidelines for aPL testing. Some were also justified for research purposes or for monitoring previously positive patients.

All appropriately categorized queries are detailed in Supplementary Table 2.

#### Group 2: unable to be evaluated

A total of 134 queries were too vague for definitive assessment, often labeled under “control.” Gynecology-related entries, such as “periconceptional exam” or “autoimmunity test,” lacked detail to confirm relevance to APS. Similarly, five cardiology-related queries citing “hypertension” could not be assessed, as testing is only justified in specific contexts like APS nephropathy. One case of “severe brain damage” might suggest thrombotic involvement but lacked clinical specificity.

### aPL-positive cohort per clinical query evaluation

When analyzing aPL-positive subjects (*n*: 115), nearly half (46.1%, 53/115) had appropriate testing requests, while 38.3% (44/115) had inappropriate testing requests, and 15.6% (18/115) could not be evaluated due to insufficient data. Regarding multiple aPL positivity, group 1 included the majority of double-, triple-, and tetra-positive cases, as shown in Table 3. When looking at the clinical query and specialist prescriber who drove aPL testing, in group 0 (inappropriate testing request) family physicians were the most common prescribers, responsible for 25/44 of these reports. Requests often involve clinical queries ranging from rheumatological and autoimmune disorders not typically linked to the syndrome, except for research purposes, to manifestations in endocrinology, gastroenterology, neurology, gynecology, and cardiology fields, not usually associated with APS. When looking at group 1 (appropriate testing request), queries included vascular and gynecological events consistent with the syndrome or rheumatic disorders commonly associated with the disease, like SLE. Family physicians and rheumatologists were the most frequent prescribers, accounting for 21/53 and 17/53 of these cases, respectively. Last, for group 2 (unable to be evaluated) family physicians were the most frequent prescribers (12/18). The most common query in this group was categorized as “control.” Details of each clinical query per group, type, and specialist prescriber are displaced in Supplementary Fig. 1.

**Table 3:** aPL-positive patients divided per number of positivity and appropriateness

aPL +	Appropriateness		
	**0**	**1**	**2**
Single	36	32	13
Double	5	11	4
Triple	2	6	1
Tetra	1	4	0

## Discussion

The study highlights significant variability in the appropriateness of aPL testing across different medical specialties, underscoring the need for evidence-based prescribing practices to optimize both diagnostic utility and resource allocation. This variability could create a ripple effect of negative consequences, impacting healthcare in multiple ways, from environmental harm to increased healthcare costs and unnecessary specialized consultations. The study cohort comprised 642 subjects, predominantly women (75.2%) with a mean age of 52 years. Of these, only 32 (4.9%) were tested for all three aPL criteria: LA, aCL, and aB2GPI. This low rate of complete testing is noteworthy and may result from two main factors: first, LA testing is often unreliable in patients on vitamin K antagonists (VKA), and in known APS patients undergoing treatment, repeat testing may be intentionally avoided; second, it may reflect a lack of awareness among requesting clinicians regarding the need for simultaneous testing of all three markers for accurate diagnosis. Furthermore, aPL positivity was detected in 17.9% of the cohort, with the majority of cases presenting as single aPL positivity (70.4%). This distribution mirrors existing literature, where single positivity is the most common pattern. However, when looking at aPL positivity in all three groups, group 1 resulted in the one with the highest rate of double, triple, and tetra positivity mirroring the appropriateness of the clinical request. Indeed, among triple and tetra aPL-positive subjects clinical query revolved around APS, SLE, and coagulopathy.

Appropriateness of testing was assessed in all 642 clinical queries: 36% were deemed appropriate, 42% inappropriate, and 22% unevaluable. Family physicians were the most frequent test requesters (53%), also showing the highest rate of inappropriate testing (44%). However, inappropriate testing was also common among specialists such as neurologists, hematologists, immunologists, and particularly gastroenterologists (86%). Rheumatologists, internal medicine physicians, gynecologists, and nephrologists demonstrated better adherence to clinical guidelines, reflecting variability in clinical training and protocol awareness.

However, it is important to consider that family physicians often serve as the first point of contact and are typically responsible for broad initial screenings. In contrast, rheumatologists and other specialists usually assess patients who have already been referred due to specific clinical concerns, leading to a higher likelihood of targeted and appropriate testing. This data can help to interpret differences in appropriateness rates across specialties and may avoid oversimplified comparisons that do not account for differences in patient population complexity and referral patterns. Yet, quantifying the number of aPL test prescriptions truly initiated by family physicians—as opposed to those for which they merely endorse or ratify a request—remains challenging.

Common inappropriate queries included conditions without strong aPL associations: oncology (e.g., cancer-related thrombosis), cardiology (e.g., aortic stenosis, tachycardia) [19], dermatology (e.g., alopecia, cellulitis) [20], endocrinology (e.g., hypercholesterolemia), gastroenterology (e.g., altered bowel function, hepatitis) [21], and gynecology (e.g., dysmenorrhea, ovarian cysts) [22–25]. While broader test panels may be justified in hospitalized patients with unclear diagnoses [26, 27], many requests lacked a clear clinical rationale or were poorly documented. This was especially evident in the 6.5% of queries mixing unrelated signs and symptoms, suggesting aPL testing was used more as exploratory rather than hypothesis-driven diagnostics. Importantly, a subset of queries categorized as inappropriate may reflect poor documentation rather than clinical irrelevance. For instance, cases labeled under “control” could involve known APS patients where the query was not explicitly stated. Conversely, appropriate indications included recurrent pregnancy loss, thrombotic events, thrombocytopenia, SLE, systemic sclerosis, and neurological conditions like stroke or transient ischemic attacks. A key issue lies in the interpretation of positive results in low-pretest-probability scenarios (e.g., isolated aCL positivity in a patient evaluated for hypotension, or tetra positivity in a patient with celiac disease) leading to unnecessary specialist referrals, repeated testing, and confusion when results normalize or prove irrelevant. While multiple positivity patterns are associated with higher thrombotic or obstetric risk, this is not absolute [14]; isolated or transient positivity may reflect inflammatory or neoplastic states rather than true APS. To address these challenges, greater adherence to diagnostic algorithms, improved clinical documentation, and targeted education, especially for non-specialists, are needed. Reducing inappropriate testing not only improves patient care but also lowers costs, conserves resources, and minimizes environmental impact [28, 29]. However, systemic barriers such as defensive medicine and unrestricted prescribing still reduce optimal test utilization. Strengths of the work lie in the detailed analysis of each query and on their evaluation. Limitations lie in the retrospective nature of the work, in the lack of testing repetition after 12 weeks, and in the lack of communication with clinicians that limit the potential appropriate evaluation of some of the vague queries. Furthermore, the heterogeneity in the number of prescriptions by specialists may lead to an underestimation or overestimation of the rate of appropriate versus inappropriate testing requests across different specialties. Similarly, one could not exclude a degree of subjectivity to the evaluation of some clinical manifestation (e.g., alopecia) as part of an autoimmune systemic disease. Finally, it was out of the scope of this very study to perform a validation exercise comparing the use of ELISA for aPL testing when comparing to other solid-phase assays.

## Conclusion

This work highlights the critical need to improve clinicians’ understanding of APS diagnostic criteria and the appropriate indications for aPL testing. By addressing appropriate aPL testing, healthcare efficiency can be ameliorated, unnecessary costs reduced, and patient care enhanced. Focusing on education, guideline adherence, and sustainability will help healthcare systems to refine diagnostic practices and to minimize resource waste and lessen its environmental impact.

## Supplementary Information

Below is the link to the electronic supplementary material. 

Supplementary file1 (DOCX 247 KB)

Supplementary file2 (DOCX 142 KB)

Supplementary file3 (DOCX 139 KB)

## Data Availability

No datasets were generated or analysed during the current study.
