# Supplementary figures and images for: Optimizing antiphospholipid antibody testing: a real-world analysis of appropriateness and resource utilization
Source: Immunol Res. 2025 Sep 16;73(1):130. doi: 10.1007/s12026-025-09682-x (PMC12441101; doi:10.1007/s12026-025-09682-x)

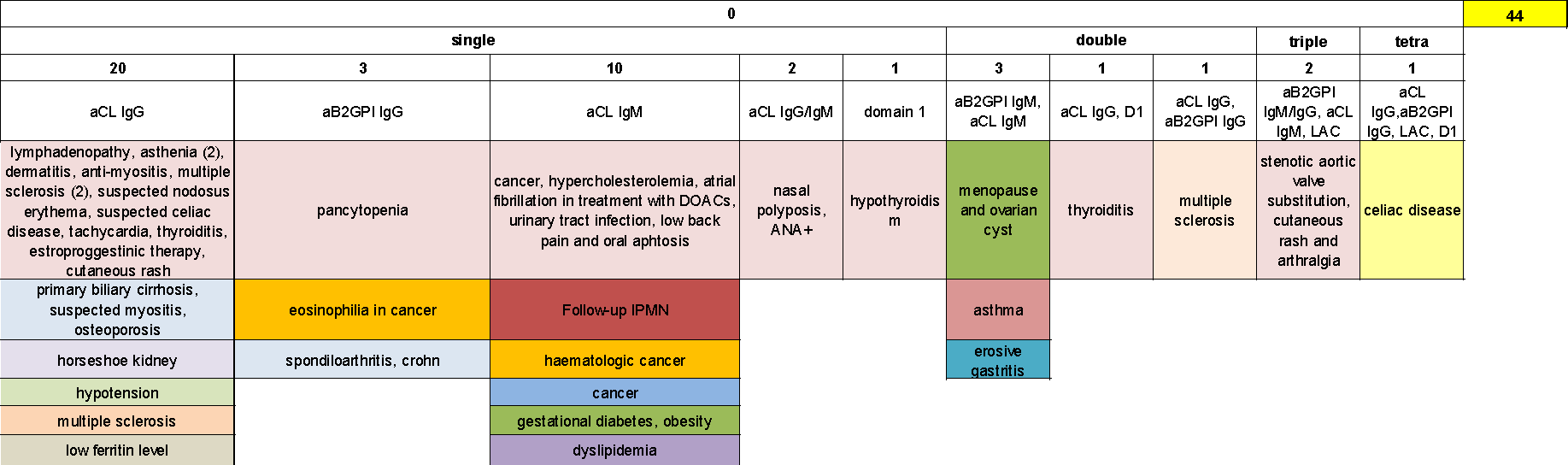


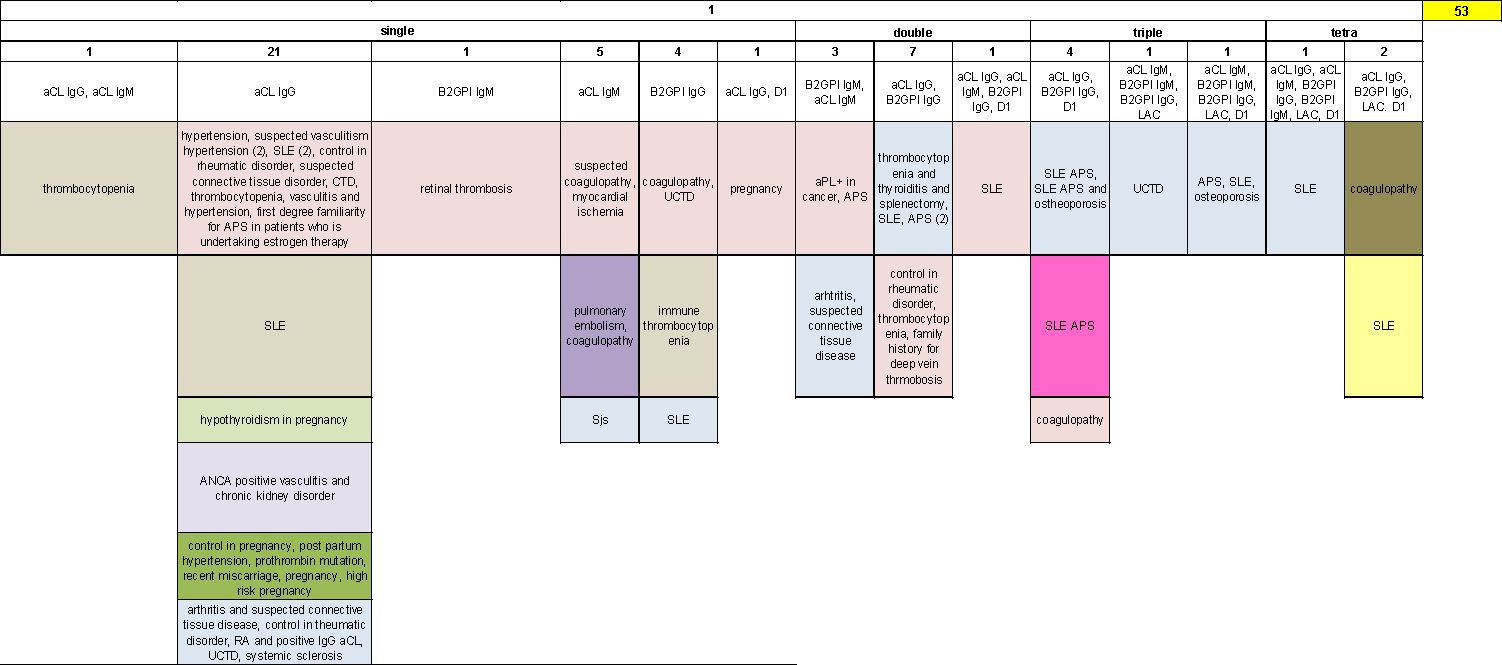


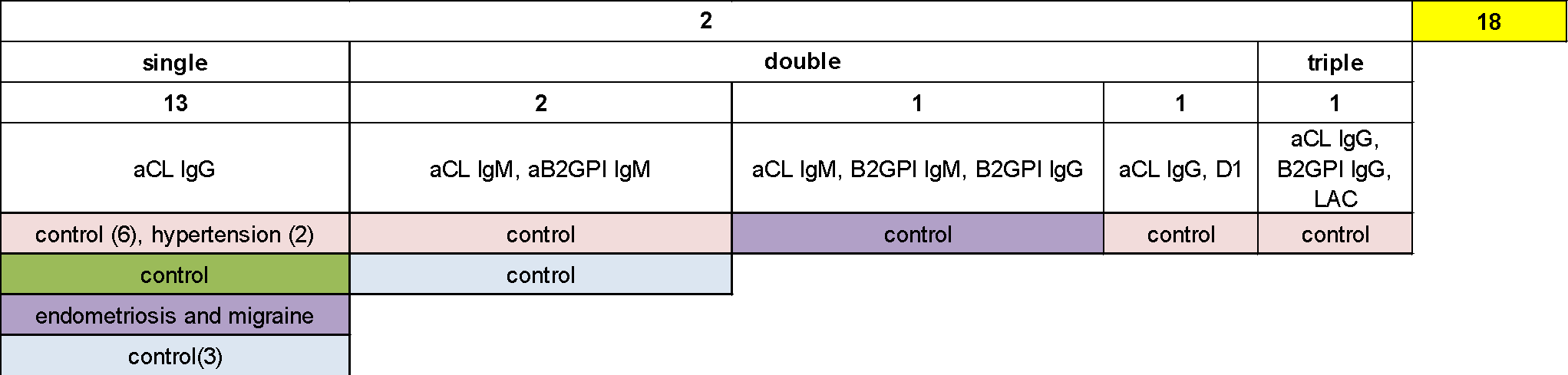


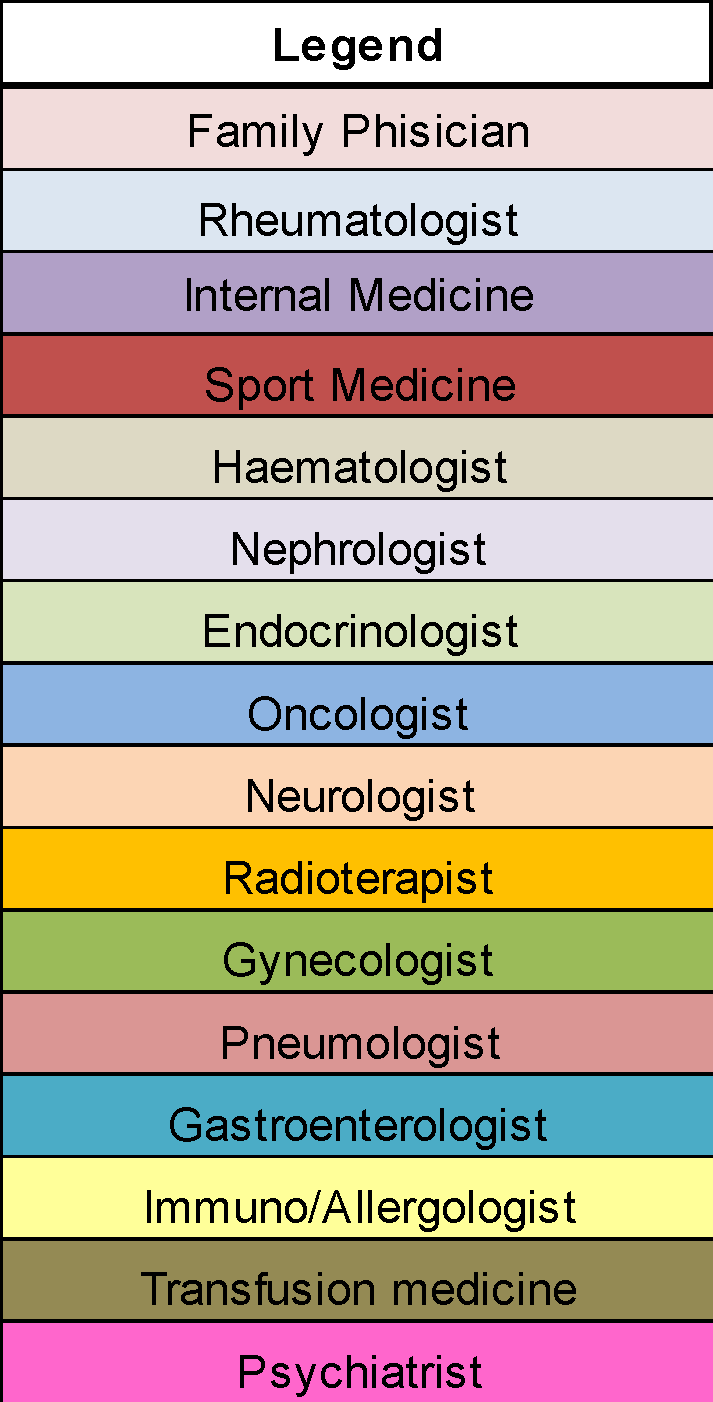


Supplementary Figure 1. Detail of clinical query for aPL positive subjects.

Supplement: Supplementary file 1 — Supplementary file1 (DOCX 247 KB) [file 12026_2025_9682_MOESM1_ESM.docx]
