# Supplementary material for: Optimizing antiphospholipid antibody testing: a real-world analysis of appropriateness and resource utilization
Source: Immunol Res. 2025 Sep 16;73(1):130. doi: 10.1007/s12026-025-09682-x (PMC12441101; doi:10.1007/s12026-025-09682-x)
Supplement: Supplementary file 2 — Supplementary file2 (DOCX 142 KB) [file 12026_2025_9682_MOESM2_ESM.docx]

| **Clinical Specialty** | **Clinical Query** | **Prescriber** | **Explanation of belonging to INAPPROPRIATE group** |
| --- | --- | --- | --- |
| Oncology (23) | Anticoagulation in cancer, cancer in association with other disorders | Family Physician, Rheumatologist, Oncologist, Internal Medicine, Pneumologist, Hematologist, Urologist, General Surgeon, Transfusion Medicine, Radiotherapist | cancer itself is a prothrombotic condition, and aPL may be present because of immune dysregulation. If anticoagulation therapy was initiated prior to the cancer diagnosis, aPL testing does not alter the therapeutic strategy. Similarly, if aPL positivity is detected after the cancer diagnosis, it does not change the overall therapeutic approach. |
| Cardiology (2) | stenotic aortic valve substitution, tachycardia | Family Physician | no correlations with APS syndrome, no strong indication sustaining aPL testing |
| Dermatology (12) | Alopecia, cellulitis, cutaneous rash, dermatitis, dorsal lipoma, migrant papulopustular lesions in the limbs, psoriasis, suspected nodosum erythema | Family Physician, Rheumatologist, Dermatologist, Geriatrician, Neurologist | no correlations with APS syndrome, no strong indication sustaining aPL testing |
| Endocrinology (36) | Diabetes, thyroiditis (suspected and confirmed), hypothyroidism, thyroid nodularity, dyslipidemia, hypercholesterolemia, hypotension, impaired fasting glucose, osteoporosis, suspected hyperglycemia, | Rheumatologist, Family Physician, Internal Medicine, Endocrinologist, Physiatrist | research purpose¸ although control of CV risk factor is important in APS, presence of hypercholesterolemia do not support screening for aPL;  no correlation with APS syndrome, no strong indication sustaining aPL testing |
| Gastroenterology (27) | altered bowel function, AMA positivity, celiac disease (suspected and confirmed, HLA predisposing gene), colon polyposis, IBD (control and suspected), Crohn. EGDS, elevated liver enzymes, erosive gastritis, follow up IPMN, HBV infection, HCV infection, high level of bilirubin, itching in chronic hepatitis, liver cirrhosis | Hematologist, Internal Medicine, Family Physician, Gastroenterologist, Allergologist/Immunologist, Rheumatologist, Sport Medicine Doctor, Infectivologist, | no correlations with APS syndrome, no strong indication sustaining aPL testing; research purpose; although aPL have been found in PBC, there is no indication on testing a patients for aPL based solely on positive AMA testing; research purpose; although HELLP and Budd-Chiari are clinical manifestations of APS, increased level of liver enzyme without high clinical suspicion for the disease are not a sufficient to sustain aPL testing; although hemolytic anemia and CAPS, for instance, can lead to increased level of bilirubin its presence do not support "per se" testing for aPL; although APS can affect the liver and cause itching, hepatitis and cirrhosis, aPL should be tested only in case of high clinical suspicion |
| Gynecology (17) | amenorrhea, anorexia, anemia and metrorrhagia, dysmenorrhea, endometriosis, estroprogestinic therapy (per sé, in sickle cell carrier), hyperinsulinemia in pregnancy, menopause, ovarian cyst, obesity and gestational diabetes, uterine fibromatosis | Family Physician, Geriatrician, Gynecologist | no correlations with APS syndrome, no strong indication sustaining aPL testing; guidelines do not sustain aPL testing in all women who are undertaking or programming to start oral contraceptive pills; lack of evidence linking sickle cell carrier status directly to a high prevalence of APS. Routine aPL testing in this patient could lead to false positives, given the relatively low pre-test probability |
| Hematology (26) | abdominal lymphadenopathy, acute lymphadenopathy, anemia, anticoagulation with DOACs (and raised level of creatinine; atrial fibrillation), elevated D dimer, factor II mutation, factor V mutation, hematologic cancer, suspected myelodysplastic syndrome, suspected polycythaemia vera, suspected MGUS, pancytopenia, protein c deficiency in a family member | Hematologist, Family Physician, Emergency Medicine Doctor, Allergologist/Immunologist, Anesthesiologist, Internal Medicine, Radiotherapist | no correlations with APS syndrome, no strong indication sustaining aPL testing; although APS can cause hemolytic anemia, anemia is a very common finding in clinical practice and can be related to multiple heterogeneous reasons. It is not a sufficient finding to call for aPL testing; If a therapeutic switch from DOACs to VKAs is being considered due to elevated creatinine levels, aPL testing does not provide additional significant justification for a decision that has already been made. Although D-dimer levels are commonly elevated in cases of active clotting and fibrinolysis, they can also increase in conditions unrelated to clot formation, such as inflammation, infection, trauma, surgery, or malignancy. Testing for antiphospholipid antibodies (aPL) based solely on elevated D-dimer levels, without clear clinical suspicion for APS, may lead to misinterpretation and false-positive results. While elevated fibrinogen levels can exacerbate the hypercoagulable state in APS, they are not specific to the condition and can occur in other inflammatory or thrombotic settings. Their presence may interfere with diagnostic tests like lupus anticoagulant assays and therefore the solely high level of fibrinogen should not sustain aPL testing, if a strong clinical suspicion is not present. In case of coagulation factor mutation aPL testing may represent a diagnostic algorithm, although apL testing in not routinely conducted in patients with coagulopathy unless with a strong clinical suspicion for the disorder. Research purpose; no indication to test for aPL in suspected myelodysplastic syndrome because diagnosis, who could support testing for risk stratification, has not been done yet. |
| Immunology/Allergology (2) | previous anaphylactic shock, nickel allergy | Family Physician | no correlations with APS syndrome, no strong indication sustaining aPL testing |
| Infectiology (1) | hiv | Rheumatologist | research purpose |
| Internal Medicine (1) | cardiac amyloidosis | Family Physician | Although amyloid raised level has been linked with APS and its manifestations, no data indicate correlation between cardiac amyloidosis and APS. |
| Mixed (39) | type II diabetes, hypertension, hypercholesterolemia, thyroiditis; alteration of inflammatory parameters; anemia and diabetes; anorexia, bulimia, estroprogestinic treatment; arthralgia, asthma; asthenia (and arthralgia); asthenia, family history for factor V mutation; contrast medium; defluvium, oligomenorrhea; diffuse pain, asthma; estrogenic therapy, acne; hypertension, dyslipidemia, benign prostatic hyperplasia, cancer; itching, arthralgia, lower limb myalgia; lower back pain and oral aphthous; lower ferritin level; lower limb edema; oligoarthritis, thyroiditis; oligomenorrhea, retinal hemorrhage during treatment with estroprogestinic; oligomenorrhea, hypertension; oral aphthous, sicca syndrome, carpal tunnel syndrome, shoulder periarthritis; prevention testing; previous hematuria, chronic hepatic disorder; primary biliary cirrhosis, cutaneous rash; psoriatic oligoarthritis, primary biliary cirrhosis; recent pregnancy cholestasis, hypothyroidism; spondyloarthritis, Crohn; suspected ulcerous colitis in diabetes mellitus; syncope, asthma; xerostomia, xerophthalmia, cancer | Family Physician, Internal Medicine, Pediatrician, Hematologist, Rheumatologist, Cardiologist | labile hypertension, dyslipidemia, lower limb edema, arthralgia are described among APS symptoms, but not sufficient per se to sustain aPL testing; although clinical manifestations of APS syndrome can sustain inflammatory blood alteration, the link is too weak to sustain appropriateness of aPL testing; no correlations with APS syndrome, no strong indication sustaining aPL testing. Arthralgia is a clinical symptom possibly suffered by APS patients, but aPL testing should not be done for investigating arthralgia; no guidelines reccomend testing all subjects with oral contraceptives for aPL. Research purpose; no guidelines indication for prevention testing. Cancer itself is a prothrombotic condition, and aPL may be present as a result of immune dysregulation. |
| Nephrology (2) | horseshoe kidney, proteinuria | Nephrologist | no correlations with APS syndrome, no strong indication sustaining aPL testing; proteinuria can be a manifestation of APS nephropathy however is not the first hint in the diagnostic algorithm and is not a good practice to test for aPL all subjects who present proteinuria. |
| Neurology (11) | multiple sclerosis (in natalizumab/teriflunomide/cladribina), myasthenia gravis | Neurologist (3), Family Physician (2) | research purpose |
| Ophthalmology (1) | sight control | Pediatrician | no correlations with APS syndrome, no strong indication sustaining aPL testing |
| Orl (2) | nasal polyposis, recurrent vertigo | Family Physician, Neurologist | no correlations with APS syndrome, no strong indication sustaining aPL testing |
| Orthopedic (2) | herniated disc, rotator cuff tear | Family Physician, Rheumatologist | no correlations with APS syndrome, no strong indication sustaining aPL testing |
| Pneumology (1) | asthma | Family Physician, Pneumologist | no correlations with APS syndrome, no strong indication sustaining aPL testing |
| Rheumatology (53) | ANA positive, anti-myositis antibodies, suspected myositis, arthralgia (suspected), suspected arthrosis, suspected autoinflammatory disease, interstitial lung disease, arthritis(suspected), oligoarthritis (PMR like), pleuropericarditis, lymphocytosis, TNF therapy, osteoporosis, cutaneous rash, drug induced SLE, fibromyalgia (suspected), asthenia, morphea, myalgia, polymyositis/dermatomyositis, psoriatic arthritis, Raynaud syndrome, rheumatic polymyalgia, suspected Horton, still disease, therapy control in rheumatic disease | Family Physician, Rheumatologist, Hematologist, Internal Medicine, | although ANA can be present in CTDs where aPL antibodies may also occur, it is relatively common and can occur in a wide range of diseases, or in case of infection, or drug intake, or in general healthy population therefore not justifying aPL testing "per se"; arthralgia, arthritis, fibromyalgia, myalgia are clinical symptoms possibly suffered by APS patients, but aPL testing should not be done for investigating these symptoms; research purpose; Osteoporosis can be linked to a lifelong treatment with VKA but is not a reason for testing patients for aPL; possible clinical association; aPL testing is not used to modify therapy if not in the very rare case of aPL negativization |
| Urology (5) | elevated PSA, post urologic surgery control, prostate hyperplasia, (recurrent) urinary tract infection | Family Physician, Urologist, Internal Medicine | no correlations with APS syndrome, no strong indication sustaining aPL testing |
| Vascular (4) | Atherosclerosis, capillary fragility, frequent ecchymosis, post-surgery control for patent foramen ovale | Family Physician, Cardiologist | accelerated atherosclerosis is described in aps, but aPL testing should not be performed in all populations presenting atherosclerosis; no correlations with APS syndrome, no strong indication sustaining aPL testing. |
