# Supplementary material for: Optimizing antiphospholipid antibody testing: a real-world analysis of appropriateness and resource utilization
Source: Immunol Res. 2025 Sep 16;73(1):130. doi: 10.1007/s12026-025-09682-x (PMC12441101; doi:10.1007/s12026-025-09682-x)
Supplement: Supplementary file 3 — Supplementary file3 (DOCX 139 KB) [file 12026_2025_9682_MOESM3_ESM.docx]

| **Clinical Specialty** | **Clinical Query** | **Prescriber** | **Explanation to belonging to APPROPRIATE group** |
| --- | --- | --- | --- |
| Oncology (1) | aPL+ in cancer | Family Physician | guidelines: aPL testing confirmation |
| Dermatology (2) | progressive cutaneous ulcers, cutaneous purpura | Family Physician | possible clinical association, diagnostic algorithm |
| Gynecology (46) | control (post) pregnancy (high risk), couple infertility, recurrent miscarriage(s), celiac disease, (control) in medical assisted reproduction, previous fetal arrhythmia, hypothyroidism, postpartum hypertension, prothrombin mutation screening, premature born, elevated liver enzyme in pregnancy, previous IUGR, medical assisted reproduction , thrombocytopenia, neurobechet in pregnancy, female infertility, first degree familiarity for APS in patient who is undertaking estrogen therapy, coagulation alteration in pregnant, autoimmune thyroiditis in couple infertility. | Family Physician, Gynecologist, Rheumatologist, Endocrinologist, Pediatrician | possible relation with previous clinical manifestations, guidelines, diagnostic algorithm, Not all miscarriages need to be investigated with aPL testing, particularly considering that celiac disorder per se represent a risk for miscarriage, diagnostic algorithm and risk stratification in case of previous pregnancy morbidity, particularly miscarriages |
| Hematology (49) | control in anticoagulation with DOACs, thrombocytopenia, thrombophilic screening, MGUS, anemia and monoclonal gammopathy, alterated gamma region at electrophoresis, DVT(previous), family history for deep vein thrombosis, elongated ptt, suspected hemolytic anemia, immune thrombocytopenia, periodical blood testing in bedridden patient, coagulopathy (suspected), cancer, previous arterial event, thrombophlebitis (recurrent), sickle cell anticoagulated with DOACs, pulmonary embolism, recurrent thrombotic events, retinal thrombosis, myocardial ischemia, hearth failure | Internal Medicine, Hematologist, Family Physician, Neurologist, Gynecologist, Clinical Pathologist, Transfusion Medicine Doctor, Cardiologist, Emergency Care Doctor, | diagnostic algorithm, possible clinical association, risk stratification, |
| Mixed (3) | thrombocytopenia, thyroiditis, splenectomy; osteoporosis, suspected thyroid dysfunction, suspected connective tissue disorder; metabolic syndrome, anemia, hepatitis, thyroiditis, thrombophilia | Rheumatologist, Family Physician, | diagnostic algorithm, possible clinical association, research purpose, osteoporosis can be cause of a long term anticoagulation, but cannot be the reason for aPL testing per sé |
| Nephrology (6) | chronic kidney disorder, control in kidney transplant | Rheumatologist, Family physician (3), Nephrologist | diagnostic algorithm, possible clinical association |
| Neurology (15) | Migraine (with aura; differential diagnosis), optic (ischemic) neuritis, epilepsy, transitory ischemic attack, stroke, previous Guillain barrè syndrome, asthenia, aphasia, brain hemorrhage | Family Physician | guidelines: aPL testing confirmation |
| ORL (1) | sudden hearing loss | Family Physician | diagnostic algorithm, possible clinical association |
| Rheumatology (107) | Suspected connective tissue disease (confirmed),  systemic sclerosis  discoid lupus erythematosus,  suspected arthritis in CTD, UCTD, SLE (HCV/HIV),  Chronic kidney disease in SLE, APS (and SLE), previous aPL positivity, autoimmune thrombocytopenia,  leukopenia and chilblain lupus,  monoclonal gammopathy and connectivitis,  (suspected) vasculitis  Vasculitis (suspected)  and hypertension,  ANCA positive (and CKD), petechiae,  polymyalgia and vasculitis, rheumatic disorder (suspected or control), rare disease (suspected or control), pericarditis in autoimmune disorder, Raynaud, fibromyalgia, arthritis,  RA positive aPL,  RA | Rheumatologist, Nephrologist, Family Physician, Hematologist, Psychiatrist, Allergologist/Immunologist Internal Medicine, Pediatrician, | diagnostic algorithm, research purpose, possible clinical association, guidelines. |
